# Strain-level characterization of foodborne pathogens without culture enrichment for outbreak investigation using shotgun metagenomics facilitated with nanopore adaptive sampling

**DOI:** 10.3389/fmicb.2024.1330814

**Published:** 2024-03-01

**Authors:** Florence E. Buytaers, Bavo Verhaegen, Tom Van Nieuwenhuysen, Nancy H. C. Roosens, Kevin Vanneste, Kathleen Marchal, Sigrid C. J. De Keersmaecker

**Affiliations:** ^1^Transversal activities in Applied Genomics, Sciensano, Brussels, Belgium; ^2^Department of Plant Biotechnology and Bioinformatics, Ghent University, Ghent, Belgium; ^3^National Reference Laboratory for Foodborne Outbreaks (NRL-FBO) and for Coagulase Positive Staphylococci (NRL-CPS), Foodborne Pathogens, Sciensano, Brussels, Belgium; ^4^Department of Information Technology, IDlab, IMEC, Ghent University, Ghent, Belgium

**Keywords:** metagenomics, nanopore, adaptive sampling, *Staphylococcus aureus*, food

## Abstract

**Introduction:**

Shotgun metagenomics has previously proven effective in the investigation of foodborne outbreaks by providing rapid and comprehensive insights into the microbial contaminant. However, culture enrichment of the sample has remained a prerequisite, despite the potential impact on pathogen detection resulting from the growth competition. To circumvent the need for culture enrichment, we explored the use of adaptive sampling using various databases for a targeted nanopore sequencing, compared to shotgun metagenomics alone.

**Methods:**

The adaptive sampling method was first tested on DNA of mashed potatoes mixed with DNA of a *Staphylococcus aureus* strain previously associated with a foodborne outbreak. The selective sequencing was used to either deplete the potato sequencing reads or enrich for the pathogen sequencing reads, and compared to a shotgun sequencing. Then, living *S. aureus* were spiked at 10^5^ CFU into 25 g of mashed potatoes. Three DNA extraction kits were tested, in combination with enrichment using adaptive sampling, following whole genome amplification. After data analysis, the possibility to characterize the contaminant with the different sequencing and extraction methods, without culture enrichment, was assessed.

**Results:**

Overall, the adaptive sampling outperformed the shotgun sequencing. While the use of a host removal DNA extraction kit and targeted sequencing using a database of foodborne pathogens allowed rapid detection of the pathogen, the most complete characterization was achieved when using solely a database of *S. aureus* combined with a conventional DNA extraction kit, enabling accurate placement of the strain on a phylogenetic tree alongside outbreak cases.

**Discussion:**

This method shows great potential for strain-level analysis of foodborne outbreaks without the need for culture enrichment, thereby enabling faster investigations and facilitating precise pathogen characterization. The integration of adaptive sampling with metagenomics presents a valuable strategy for more efficient and targeted analysis of microbial communities in foodborne outbreaks, contributing to improved food safety and public health.

## 1 Introduction

Investigating bacterial foodborne outbreaks involves identifying the source of contamination to prevent further spread and ensure public health. The illness can be caused by the microorganism itself (e.g., *Salmonella*), or by the toxins produced by the microorganism (e.g., *Bacillus cereus, Staphylococcus aureus*). In both cases, the investigation focuses on determining the food source responsible for the outbreak. This is done through a combination of epidemiological investigations and laboratory analysis. However, in 60% of foodborne outbreaks notified in the EU, the food source cannot be determined with certainty (EFSA, 2021). This can be explained in part by the lack of leftover food to test. However, it can also partially be due to a gap in detection with the current practices. Traditionally, food samples suspected of causing contamination are subjected to enrichment procedures, which involve incubating the sample in a medium that supports the growth of the target bacteria. While this increases the total turnaround time of the analysis, this step allows the bacteria to multiply and reach detectable levels before further analysis. It can be followed by isolation of the pathogenic bacterium which can then be characterized and compared to the human cases (EFSA, 2019). However, as previously reported, a bias can occur during the enrichment of the food due to competition between the various living organisms or the cultivation parameters such as temperature (Jasson et al., 2009, 2011). Several studies have also established the challenge to obtain an isolate from complex food matrices (Gupta et al., 2015; Foddai and Grant, 2020). When the isolation is not obtained, the pathogen is not fully characterized and due to lack of microbiological evidence, the outcome of the outbreak investigation is labeled as “weak” (EFSA, 2014).

Shotgun metagenomics is a sequencing method involving the analysis of the genetic material directly extracted from a sample, without the need for an isolation of the bacteria. By sequencing and analyzing the DNA of the sample, it can identify the presence of various pathogens to the strain level and detect genes associated to antimicrobial resistance, virulence or production of toxins (Leonard et al., 2015; Forbes et al., 2017; Buytaers et al., 2020). Therefore, this approach allows for an efficient detection of the causative agent of the outbreak, even in cases where the exact pathogen is unknown or unexpected (Kawai et al., 2012; Miller et al., 2013). Additionally, it has been shown to perform this evidence-based outbreak investigation of the food in a faster time-frame than the current methods (Buytaers et al., 2021b,c). However, the detection of the pathogen with shotgun metagenomics was reported as being less sensitive compared to a polymerase chain reaction (PCR) test, and therefore the enrichment of the food sample was still required in order to increase the contamination load (Leonard et al., 2016; Buytaers et al., 2020). Alternatively, when no culture enrichment is possible, e.g., for viral foodborne pathogens, a whole genome amplification of the DNA extracted from the sample has been previously investigated (Buytaers et al., 2022).

Oxford Nanopore Technologies (ONT) sequencing is a next-generation sequencing technology that utilizes nanopores to directly sequence DNA or RNA molecules. It offers several advantages, such as long read lengths and real-time data streaming and analysis. A new feature that has been recently implemented with ONT sequencing is the adaptive sampling (ONT AS, Cheng et al., 2022; Martin et al., 2022). This allows to sequence preferentially DNA strands that are similar to those presented in a database. It has previously been used to selectively deplete for known host DNA, e.g., the matrix such as human DNA (Marquet et al., 2022; Marchukov et al., 2023) or to selectively enrich for DNA of interest, i.e., the pathogen, e.g., foodborne viruses which cannot be cultured (Buytaers et al., 2022).

In this study, we explored the combination of whole genome amplification, adaptive sampling sequencing and shotgun metagenomics in an attempt to show the feasibility of a foodborne bacterial outbreak investigation without culture enrichment nor isolation. First, shotgun metagenomics sequencing was compared to ONT AS with different databases on DNA extracts of potatoes mixed with DNA of a *S. aureus* strain previously linked to an outbreak (Nouws et al., 2021). Second, the performance of three DNA extraction kits was compared on potatoes spiked with the living pathogen, without enrichment. The contamination load was 10^5^ CFU in the food sample (25 g), a representative contamination level in a natural situation. The information obtained after metagenomics analysis was compared to the information from whole genome sequencing of the isolated pathogen. We propose the most informative workflow to determine the presence of a pathogen, its potential to produce toxins and relate it in case of outbreak while bypassing the need for enrichment and isolation, and therefore enabling quicker identification and characterization of the causative agent.

## 2 Materials and methods

### 2.1 *Staphylococcus aureus* culture preparation

The *Staphylococcus aureus* strain TIAC 1798 from the collection of the Belgian National Reference Laboratory (NRL) was used for this study. It was isolated from potatoes in 2013 and was the source of an outbreak with several human cases. Using conventional methods, TIAC 1798 was found to produce the enterotoxin A (SEA). The presence of the staphylococcal enterotoxin A encoding gene, *sea*, in addition to other enterotoxin encoding genes, was confirmed using isolate whole genome sequencing (Nouws et al., 2021).

The strain TIAC 1798 was cultivated from the glycerol stock of the collection on nutrient agar for 24 h at 37°C. One colony was inoculated in 10 ml of brain heart infusion broth (BHI), and the liquid culture was incubated for 16 h at 37°C. The culture was then diluted in BHI to obtain an OD600 of 1. A counting was performed in triplicate on nutrient agar after making serial dilutions using buffered peptone water (BPW). The counting indicated that the OD600 = 1 culture contained 1.25 × 10^8^ CFUs/ml.

### 2.2 Artificial contamination of mashed potatoes

Pre-bagged mashed potatoes bought at a local store (ingredients: 68% potatoes, semi-skimmed milk, 4% butter, salt, white pepper, nutmeg, curcuma extract, emulsifier: mono- and diglycerides of fatty acids) were divided in portions of 25 grams. Each portion was diluted in 225 ml BPW and homogenized by stomaching as described previously (Buytaers et al., 2020). One portion was kept as such (the “blank”). One portion (25 grams of mashed potatoes in 225 ml BPW) was contaminated with TIAC 1798 with a level of 10^5^ CFUs. This corresponds to ∼450 CFUs per milliliter used for DNA extraction. The bags were homogenized by hand massage and aliquots of these spiked and blank mashed potatoes were sampled. Subsequently, the bag of the blank was incubated at 37°C for 24 h. After incubation, aliquots were sampled as well. A total of 1 ml of each liquid mix were centrifuged at 6,000 g for 10 min and the cell pellets were stored at −20°C until DNA extraction.

### 2.3 DNA extract preparation

The DNA of the isolated cultured strain (TIAC 1798) was extracted in a previous study (Nouws et al., 2021) using the Genelute Bacterial gDNA kit (Sigma-Aldrich, Missouri, United States) following the specific protocol for *Staphylococcus* species (with the use of lysostaphin).

The DNA of the cell pellets from 1 ml of blank (enriched or not) and spiked mashed potatoes was extracted using Nucleospin Food (abbreviated as “N,” Macherey-Nagel, Düren, Germany) as previously described (Buytaers et al., 2021b). For the spiked sample, HostZERO Microbial DNA kit (abbreviated as “HZ,” Zymo Research, Irvine, CA, USA) in order to remove the eukaryotic DNA of the sample and selectively extract DNA of the microorganisms, and Quick-DNA HMW MagBead Kit (abbreviated as “Q,” Zymo Research, Irvine, CA, USA) that is described as allowing to obtain longer DNA fragments for further long-read sequencing and adaptive sampling (Martin et al., 2022) were also tested. All kits were used according to the manufacturer’s recommendations.

An artificial DNA preparation was also prepared by mixing 0.5% of DNA from *S. aureus* isolate DNA TIAC 1798 (Nouws et al., 2021) with 99.5% DNA of blank mashed potatoes enriched for 24 h (to harbor background bacteria). This mix was prepared in order to be in the optimal range for the use of adaptive sampling (as recommended by the manufacturer, i.e., 0.5% of “target” DNA). This mix corresponds to a contamination of ∼10^9^ genome copies of *S. aureus* in 25 g of potatoes after calculation. As a positive control for the qPCR, the same quantity of *S. aureus* was spiked in water.

The DNA concentration was measured with a Qubit 3.0 Fluorometer (Thermo Fischer Scientific, Waltham, MA, USA) and the presence of *S. aureus* in the DNA extracts was evaluated with a qPCR test of the *sea* gene (Paule et al., 2004). This gene was selected as target for the qPCR test because the spiked strain (TIAC 1798) was described as producer of the enterotoxin A and because the presence of the gene *sea* was confirmed using WGS data of the isolated strain (Nouws et al., 2021). The qPCR test was also conducted on the blank potatoes and the blank potatoes enriched for 24 h in order to verify the absence of *S. aureus* in the matrix.

### 2.4 Library preparation and sequencing

The libraries were prepared with two different protocols depending on the samples: the DNA mix was sequenced using the ligation sequencing kit (SQK-LSK109 and SQK-LSK110; Oxford nanopore Technologies, Oxford, United Kingdom). The libraries of the potatoes spiked with the living bacteria were prepared according to the whole genome amplification ligation (SQK-LSK109 and SQK-LSK110) sequencing protocol from Oxford nanopore Technologies with some modifications: the DNA was amplified for 16 h using the REPLI-g Midi kit (Qiagen, Hilden, Germany). At this point, the amplified DNA was tested for concentration and detection of the *sea* gene by qPCR (Table 1) as described above (Paule et al., 2004). The amplified DNA was then cleaned using AMPure XP beads. T7 endonuclease (NEB, Ipswitch, MA, USA) was used to cleave single-stranded DNA as well as four-way junctions (branches). A cleaning was conducted using the Monarch PCR & DNA Cleanup Kit (NEB, Ipswitch, MA, USA). Approximatively 700 ng of DNA was then used for the DNA repair, end-prep and ligation. All libraries were loaded on R9.4 flow cells (one sample per flow cell) and a 72 h run was performed on a GridION device. ONT AS was used for some sequencing, selectively depleting for the potato genome (samples_potato, based on the reference of the 12 chromosomes of *Solanum tuberosum cultivar Solyntus*, GenBank CP055234.1 to CP055245.1 provided as one mmi file), enriching for *S. aureus* genomes (samples_saureus, based on the concatenation of all contigs longer than 1000 bp of assemblies from all isolates from Nouws et al. (2021) as well as *Staphylococcus aureus* subsp. *aureus* NCTC 8325 chromosome, complete genome (Refseq NC_007795.1), provided as a mmi file) or enriching for foodborne pathogens genomes (samples_foodpath, based on a selection of reference genomes of several foodborne genomes including one *S. aureus* (Refseq NC_007795.1), fasta file provided as Supplementary Material 1, the file was provided as a mmi). The sequencing of the DNA mix with shotgun metagenomics and of the three different DNA extracts with ONT AS using the database containing *S. aureus* was repeated (sample_shotgun2 and samples_saureus2, respectively).

**TABLE 1:** Detection of the *sea* gene for the production of enterotoxin A by qPCR in different samples prepared by mixing DNA extracted from mashed potatoes and an *S. aureus* isolate.

Sample	Mean Cq value ± standard deviation
Blank	ND
Blank enriched 24 h	ND
positive control *S. aureus*	22.05 ± 0.05
DNAmix	23.08 ± 0.03

Blank = DNA extracted from the matrix (mashed potatoes); Blank enriched 24 h = DNA extracted from the matrix (mashed potatoes) enriched for 24 h; Positive control *S. aureus* = positive control (mix of DNA extracted from *S. aureus* isolate and water)*;* DNAmix = mix of DNA from blank mashed potatoes enriched for 24 h and DNA of isolated *S. aureus* TIAC 1798. qPCR Cq *sea* = indicative for the presence of the spiked TIAC 1798 strain. ND: no detection occurred after 40 cycles.

### 2.5 Data analysis

After basecalling using guppy version 5.0.7, statistics were obtained on the reads with NanoStat (De Coster et al., 2018). For the ONT AS, BBmap version 38.34 (Bushnell, 2014) was used to retain only the reads “stop receiving” based on the adaptive sampling csv file (samples_stop) as opposed to keeping all the sequenced reads (samples_all). The stop_receiving reads are the DNA molecules that were accepted for sequencing through the pore, and therefore were sequenced entirely (as opposed to the unblock reads which were rejected). All sequenced reads comprise the stop_receiving reads, the unblock reads (the small parts of the DNA molecules that were sequenced before releasing of the DNA) and some “no decision” reads. Because the reads were divided in stop or all reads, only a limited filtering was conducted on all the sequenced reads in order to maintain the difference: NanoFilt was used to filter out all reads smaller than 300 bp and with a phred value < 7 (De Coster et al., 2018). A taxonomic classification of the reads was conducted using Kraken2 (Wood et al., 2019) with the same in-house database of mammals, archaea, bacteria, fungi, human, protozoa and viruses as described in Buytaers et al. (2022). The percentage of unclassified reads and reads classified as *S. aureus* was calculated. The presence of enterotoxin encoding genes was tested using Blastn version 2.13.0 (Camacho et al., 2009) with default parameters on the database described by Nouws et al. (2021). The gene was considered detected as from 1 hit. Finally, the reads corresponding to *S. aureus* were obtained by using Metamaps (Dilthey et al., 2019), as downloaded from Github on April 7th, 2023 with the RefSeq (O’Leary et al., 2016) database of bacteria (downloaded on 30/11/2022). All reads mapping to any *S. aureus* reference were considered as the *S. aureus* strain in the sample and used for further analysis on this strain. These reads were further characterized with a Blastn search (Camacho et al., 2009) on the same database of enterotoxin genes. Single nucleotide polymorphism (SNP) calling on the metagenomics-derived reads corresponding to the *S. aureus* strain was conducted using bwa mem version 0.7.17 (Li, 2013) with the ont2d parameter for ONT sequences as described in Buytaers et al. (2021b) and on Illumina isolates’ data from Nouws et al. (2021) with SMALT version 0.7.6 (Ponstingl and Ning, 2010) and SAMtools version 1.9 (Li et al., 2009) as described in Saltykova et al. (2020) with the reference *S. aureus* subsp. *aureus* NCTC 8325 chromosome, complete genome (RefSeq NC_007795.1). The isolates for which the Illumina data were used for the phylogenetic analysis (Nouws et al., 2021), were the spiked strain (TIAC 1798), other isolates linked to TIAC 1798 in a previously resolved outbreak (TIAC 1847, TIAC1848) and unrelated cases (TIAC 1840, 1991, 1992, 1993, 1994, 2001, 3152, 3462, 3462, 3972). Blastn was used on the classified reads with the same database and parameters as for all the reads to detect the presence of the enterotoxin encoding genes at strain level. Bcftools version 1.9 (Li, 2011) was then used for the identification and masking of the SNPs in the consensus sequence with the same parameters as used previously (Buytaers et al., 2021b). The phylogenetic tree was constructed after automatic model selection using MEGA (Kumar et al., 2018) applying the nearest-neighbor-interchange (NNI) heuristic method, keeping all informative sites and using the bootstrap method with 100 replicates. The model selected was General Time Reversible and the tree representation was made using iTOL v5 (Letunic and Bork, 2021), adding the percentage of the reference covered and the detection of several enterotoxin encoding genes to the side of the tree.

## 3 Results

### 3.1 Comparison of different adaptive samplings and shotgun metagenomics sequencing

A mix of DNA from the isolated *S. aureus* strain TIAC 1798 and DNA extracted from mashed potatoes enriched for 24 h was prepared. The presence of *S. aureus* DNA was confirmed by qPCR (Table 1) in this spiked sample (DNAmix) and had a similar Cq to the positive control, confirming the absence of matrix effect. The mashed potato matrix was free of *S. aureus* contamination prior to the experiment, as determined by qPCR analysis of the blank and the blank enriched for 24 h (Table 1). The spiked sample (*S. aureus*-potato DNA mix) was sequenced four times on a GridION device, once with shotgun metagenomics, once with adaptive sampling depleting the DNA similar to a reference sequence of potato (*Solanum tuberosum*, GenBank CP055234.1), once with adaptive sampling enriching for the DNA similar to several references of *S. aureus* and once with adaptive sampling enriching for the DNA similar to references of various foodborne pathogens, including one reference for *S. aureus* (Supplementary Material 1).

#### 3.1.1 Detection of the spiked pathogen using taxonomic classification after adaptive sampling or shotgun metagenomics sequencing

A taxonomic classification was conducted on all the reads and, in the cases of ONT AS, also only on the reads that were tagged “stop receiving” instead of all the sequenced reads (Table 2; Supplementary Material 2). The most represented genus in all samples was *Paenibacillus*, a genus naturally present in the environment and previously reported on potatoes (Liu et al., 2016; Yahyaoui et al., 2023), which covered over 70% of the reads (Supplementary Material 2), and this was not affected by using the stop_receiving reads for enrichment AS, but gained 10% when depleting for the potato genome. The genus *Bacillus*, which can also correspond to a foodborne pathogen, was also detected at low percentages in all samples (Supplementary Material 2). However, *Bacillus cereus* was not reported at species level. *Klebsiella* and *Escherichia* were also reported by the taxonomic classification tool once each at very low percentages (0.02% and 0.01%) in the stop_receiving reads, but were probably false positives. Finally, some reads were misclassified as *Homo* (human), *Rattus* (rat) or *Mus* (mouse), in particular in the stop_receiving reads after depletion of the potato genome. The only pathogen, detected at levels higher than 0.1%, was the spiked pathogen (*S. aureus*) (Table 2; Supplementary Material 2). When comparing for all the sequenced reads, the results are similar with or without ONT AS and irrespective of the database used: *S. aureus* was detected as the pathogen in the samples at approximatively 0.1%, while it was spiked at 0.5%. Seventeen to 76% of the reads were unclassified (potentially representing the extracted DNA from the potatoes, a species that is not present in the database used for the taxonomic classification). The very high amount of unclassified reads in the second replicate of the shotgun sequencing has been investigated by using an alternative classification method: all reads were Blasted to the ncbi database as previously described (Buytaers et al., 2021a). The reads were mainly classified as *Paenibacillus* and *Bos mutus* (which was not present in the Kraken database).

**TABLE 2:** Detection of *S. aureus* by taxonomic classification and proportion of unclassified reads in all reads or stop receiving reads after shotgun metagenomics sequencing or adaptive sampling with different databases.

	All reads					Stop_receiving reads				
Sequencing	The number (of) reads	Median read length	*% S.aureus*	*The number (of) S.aureus* reads	% unclassified	The number (of) reads	Median read length	*% S.aureus*	*The number (of) S.aureus* reads	% unclassified
shotgun	612,328	969	0.1	611	19.02					
shotgun2	3,512,279	1036	0.03	1105	76.4					
AS potato	17,159,215	1229	0.1	4195	17.73	480,228	6329	0.18	856	2.68
AS *S. aureus*	5,123,713	476	0.1	5220	22.14	22,980	1281	20	4592	0.06
AS Foodpath	2,771,148	478	0.07	2094	24.85	11,784	1,368	14.8	1710	0.04

stop_receiving reads = reads that did not match (depletion) or matched (enrichment) the reference sequences provided in the database. Shotgun: shotgun metagenomics sequencing. AS potato: Adaptive sampling depleting for the potato genome. AS *S. aureus*: adaptive sampling enriching for a database of *S. aureus* genomes. AS foodpath: adaptive sampling enriching for a database of foodborne pathogens genomes, including *S. aureus*.

When looking at the stop_receiving reads, the amount of unclassified reads dropped drastically for the depletion of potatoes and for the enrichment of *S. aureus* or food pathogens, supporting the suggestion that the unclassified reads when considering all sequenced reads mainly represented the matrix (potato being absent from the Kraken database used). For the depletion of the potato DNA, the detection of *S. aureus* was almost doubled, while after targeting *S. aureus* the detection reached 20% of the reads and 14.8% with an AS database representing various foodborne pathogens. However, the number of reads classified as *S. aureus* was lower than when considering all reads of the same sequencing although longer in median read length (Table 2).

#### 3.1.2 Characterization of the spiked pathogen: detection of the enterotoxin encoding genes after adaptive sampling or shotgun metagenomics sequencing

**FIGURE 1:**
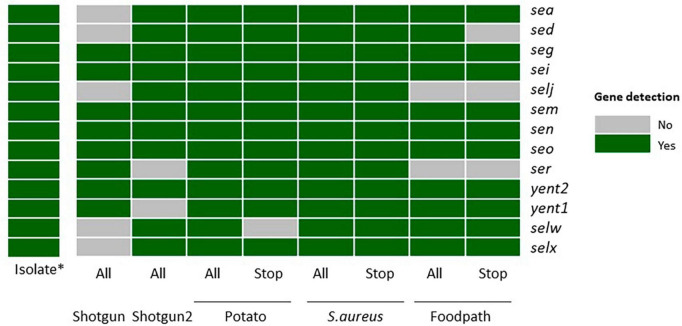
Detection of the enterotoxin encoding genes specific to strain TIAC 1798 in metagenomics samples with or without adaptive sampling on all sequenced reads or only the reads classified as “stop_receiving” gray: not detected. green: detected. *: data from Nouws et al. (2021). All: all sequenced reads; Stop: “stop_receiving” reads, or reads that mapped the AS database (in case of enrichment) or did not map the AS database (depletion of potato); Shotgun: shotgun metagenomics sequencing (no AS), Potato: AS depleting for potato DNA; *S. aureus*: AS enriching for a selection of *S. aureus* genomes, Foodpath: AS enriching for a selection of genomes of foodborne pathogens.

The enterotoxin encoding genes specific to the strain TIAC 1798 have been determined on the isolate by Nouws et al. (2021). These have been compared to the toxin encoding genes detected after shotgun metagenomics or ONT AS, on all reads or the stop receiving reads (Figure 1).

The first shotgun metagenomics sequencing detected the lowest number of genes (8/13) while the replicate (with more total reads obtained, Table 2) missed two genes. Adaptive sampling depleting the potato DNA detected all expected enterotoxin encoding genes in all reads, but one gene (*selw*) was lost if looking at the stop receiving reads. Adaptive sampling enriching compared to a database of *S. aureus* allowed to detect all genes in all reads or in the stop receiving reads. Finally, two genes (*selj, ser*) were missing when enriching with a database of reference genomes from foodborne pathogens, and three (*sed*, *selj, ser*) if testing only the stop receiving reads.

In view of these results, although the “stop_receiving” reads presented higher proportions of the pathogen of interest (Table 2), some information (genes) was lost compared to all of the sequenced reads, except when using the *S. aureus* database (Figure 1). This might be explained by the drop in the number of “stop_receiving” reads classified as *S. aureus* (Table 2). The difference in total reads sequenced between the replicates of shotgun metagenomics (612,328 and 3,512,279) explains the difference in the number of genes detected in these two samples.

Therefore, all the reads from ONT AS were taken into account for the strain-level analysis instead of just the “stop_receiving” reads. The adaptive sampling still plays a role as compared to shotgun metagenomics without AS even when considering all the sequenced reads after AS sequencing, as the *S. aureus* reads should be longer than the other reads and therefore carry more genetic information (presence of genes) due to adaptive sampling.

#### 3.1.3 Strain-level analysis and phylogeny of the spiked pathogen after adaptive sampling or shotgun metagenomics sequencing

**FIGURE 2:**
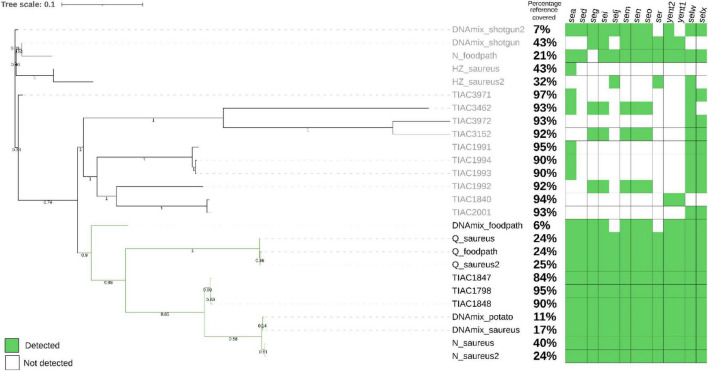
Phylogenetic tree after SNP calling on the metagenomics derived strains after shotgun metagenomics or adaptive sampling sequencing as well as the isolate that was spiked (TIAC 1798), other isolates linked to TIAC 1798 in a previously resolved outbreak (TIAC 1847, TIAC1848) and unrelated cases (TIAC 1840, 1991, 1992, 1993, 1994, 2001, 3152, 3462, 3462, 3972). For metagenomics samples, the name is composed of the sample name (DNAmix for the first experiment, mix of *S. aureus* and mashed potatoes DNA) or the kit used (N for Nucleospin Food, H for HostZERO, Q for Quick DNA HMW) for the second experiment (DNA extracted from matrix spiked with living *S. aureus*) (cfr. 3.2), and the database used for adaptive sampling (_potato, _foodpath, _saureus or _saureus2 for the repeat). Black: outbreak cluster. The percentage of the reference covered by the reads and the detection of enterotoxin genes (green: present, white: absent) are presented on the side of the tree. The scale bar represents nucleotide substitution per nucleotide site. Values on the branches represent bootstrap support values. The strains obtained from stop_receiving reads of the spiked DNA and after HostZero extraction and ONT AS using the foodpath database could not be placed on the tree as all SNPs were masked.

A strain-level analysis was performed by mapping all reads of the metagenomics samples to a database of bacterial reference genomes. All reads mapping to *S. aureus* references were retained for further analysis as being the *S. aureus* strain in the metagenomics sample. SNP calling was performed on those strains as well as the isolate that was spiked (TIAC 1798), other isolates linked to TIAC 1798 in a previously resolved outbreak (TIAC 1847, TIAC 1848) and unrelated cases (other TIAC). A phylogenetic tree was made based on this information (Figure 2; Supplementary Material 3). Gene detection in all isolates and in *S. aureus* strains from metagenomics samples was also conducted on the inferred strains and isolates.

The *S. aureus* strains from DNA spiking of the pathogen were placed in the outbreak cluster in the phylogenetic tree only after using ONT AS with the databases for potato (DNAmix_potato) and *S. aureus* (DNAmix). The strains contained all enterotoxin encoding genes from the spiked isolate (Figure 2). The strain obtained after ONT AS using a database of various foodborne pathogens (DNAmix_foodpath) was placed in the outbreak cluster but on a separate branch. This strain presented a low percentage of coverage of the reference (6%) although 11 out of 13 enterotoxin encoding genes were detected.

However, the strains obtained after shotgun metagenomics sequencing (DNAmix_shotgun and DNAmix _shotgun2) were placed outside the outbreak cluster (Figure 2). Moreover, the inferred strains after shotgun metagenomics sequencing were missing several enterotoxin encoding genes compared to the spiked strain (TIAC 1798).

### 3.2 Evaluation of different DNA extraction kits for the characterization of a foodborne pathogen in a food sample without enrichment using adaptive sampling

Oxford Nanopore Technologies AS showed its added value over shotgun metagenomics on DNA of potatoes spiked with DNA of a pathogen. However, this is corresponding to a relatively high contamination level (qPCR Cq of 23, Table 1) and it did not include the DNA extraction step from the contaminated food sample. In order to test the same method on a more realistic situation, different DNA extraction kits were tested on mashed potatoes artificially spiked with a strain of *S. aureus* (TIAC 1798) at a level of 10^5^ CFUs per 25 g of mashed potatoes. The sample was properly mixed and then immediately extracted without prior enrichment using three extraction kits: Nucleospin Food (N), HostZERO (HZ) and Quick-DNA HMW Magbead (Q). The DNA extract was then amplified using whole genome amplification to obtain more genetic material. With this amplification, the contamination reached *S. aureus* levels in the range of the DNA mix sample, as demonstrated by qPCR (Tables 1, 3).

**TABLE 3:** Detection of the *sea* gene for the production of enterotoxin A by qPCR in the different metagenomics samples prepared with or without DNA amplification.

Sample		Mean Cq value ± standard deviation
Nucleospin	DNA	36.59 ± 0.13
	amplified DNA	22.14 ± 0.24
Hostzero	DNA	37.04 ± 0.6
	amplified DNA	28.08 ± 0.16
Zymo quick	DNA	33.91 ± 0.28
	amplified DNA	25.62 ± 0.23

qPCR Cq *sea* = indicative for the presence of the spiked TIAC 1798 strain. Nucleospin = Mashed potatoes spiked with *S. aureus* extracted with Nucleospin Food; Hostzero = Mashed potatoes spiked with *S. aureus* extracted with HostZero; Zymo Quick = Mashed potatoes spiked with *S. aureus* extracted with Zymo quick HMW. DNA: immediate testing of the DNA extract. Amplified DNA: qPCR test after DNA amplification.

The amplified DNA was sequenced using ONT AS with a database of foodborne pathogens (a more open approach) or a database of *S. aureus* (a targeted approach when the pathogen is known). The database depleting for the matrix (potato) was not used as the results (Figure 2 and Table 2) were not better than the use of the *S. aureus* database and it would not be practical in a routine setting to know the exact composition of each matrix and set up a database for each sample. The performance of the two AS databases was compared for their ability to detect the pathogen spiked in the sample without enrichment and to characterize it to the strain-level.

#### 3.2.1 Detection and identification of the foodborne pathogen

After taxonomic classification, *S. aureus* was detected as the only pathogen in all samples (Table 4; Supplementary Material 2) as no *Bacillus cereus* could be detected at species level. In the HostZero DNA extracts, *Klebsiella* and *Salmonella* had hits in the taxonomic classification output at low percentages and *Escherichia* was detected at 15 to 27%. This DNA extraction kit also led to a higher detection of *Viridibacillus* but a reduction of the detection of *Paenibacillus*, the main genus reported in all other samples, by at least 40%. *S. aureus* was also detected with the highest level after DNA extraction using the HostZERO kit (between 9 and 12% compared to 0.1% and less for the other DNA extraction kits) and with slightly higher detection when using the *S. aureus* database for ONT AS.

**TABLE 4:** Detection of *S. aureus* and proportion of unclassified reads in all reads after adaptive sampling using different databases for ONT AS.

Adaptive sampling database	DNA extraction kit	total number of sequenced reads	*% S.aureus*	*The number (of) S.aureus reads*	% Unclassified
Foodpath	Nucleospin food	2,358,730	0.11	2,524	19.59
	HostZero	10,510,559	9.45	992,759	12.37
	Quick DNA HMW	10,476,802	0.05	4,873	36.2
*S.aureus*	Nucleospin food	9,158,289	0.1	8,715	20.48
		6,490,648	0.1	6,676	20.74
	HostZero	2,087,856	12.26	256,051	17.4
		5,132,465	11.01	565,158	13.57
	Quick DNA HMW	10,633,668	0.05	5,460	35.53
		13,538,475	0.05	6,391	35.93

The samples extracted with the Nucleospin kit or the Quick-DNA HMW kit both had low levels of *S. aureus* classified reads regardless of the database for ONT AS (Table 4). The amount of unclassified reads was over ten percent higher after extraction with the Quick-DNA HMW kit compared to the other DNA extraction methods (Table 4), however this kit also led to a higher number of sequenced reads.

#### 3.2.2 Strain-level characterization and phylogeny

The reads mapping to *S. aureus* references using Metamaps were further analyzed as the pathogen’s strain within the sample. A gene detection was conducted on those reads and a phylogenetic tree was constructed with other *S. aureus* isolates (Figure 2).

After ONT AS using the foodpath database, the strain obtained after extraction with HostZero could not be included in the tree because all SNPs were masked during the analysis. The strain obtained from the Nucleospin Food extract (N_foodpath) was not correctly placed in the phylogenetic tree and was missing one enterotoxin encoding gene compared to the spiked isolate (TIAC 1798). However, the strain obtained after extraction with the Quick DNA HMW kit was placed close to the spiked isolate within the outbreak cluster and harbored all the expected genes.

ONT AS using a database of *S. aureus* genomes was repeated to establish repeatability and yielded similar results: the strains obtained after extraction with the HostZERO (H_saureus) were not placed correctly on the tree and presented only partial genes present in TIAC 1798. The strains obtained from the sample extracted using the Quick-DNA HMW kit were placed in a separate branch within the outbreak cluster (Figure 2) along with the same DNA extract sequenced using the foodborne database, and these strains contained all genes detected in TIAC 1798. Finally, the strains obtained from the sample extracted with Nucleospin Food were placed closely to the outbreak isolates, which were sequenced with Illumina. These two strains also contained all genes present in the outbreak isolates and covered 40% and 24% of the reference genome while the isolates from the outbreak covered between 84 and 95% of the reference genome.

## 4 Discussion

The use of shotgun metagenomics has already proven its positive impact for the investigation of bacterial foodborne outbreaks, by providing more comprehensive and rapid insights into the microbial communities associated with outbreaks (Huang et al., 2017; Buytaers et al., 2021c; Grützke et al., 2021). However, culture enrichment was still a necessary step to have sufficient contamination load in the matrix prior to sequencing (Leonard et al., 2015; Hyeon et al., 2018; Buytaers et al., 2020). In this work, we investigated how the use of ONT AS combined with DNA amplification after normal DNA extraction or eukaryotic DNA depletion could complement the existing work on metagenomics for the study of foodborne contaminations and foodborne outbreaks without the need for culture enrichment, by providing a more targeted approach during the sequencing.

First, as a proof of concept of the added value of ONT AS, sequencing with different databases was conducted on DNA extracted from an isolated *S. aureus* linked to an outbreak (Nouws et al., 2021) added to DNA extracted from mashed potatoes, with the goal first to detect the pathogen and second to characterize it, i.e., to uncover its potential to produce toxins and to determine the ability to perform phylogenetic analysis. This was compared to a shotgun metagenomics sequencing. All sequencing methods allowed to detect the pathogen. Using the subset of reads that mapped to the database during the ONT AS sequencing, i.e., the stop_receiving reads (Marquet et al., 2022), increased the proportion of *S. aureus* detected as the pathogen in the sequencing compared to the detection in all sequenced reads, hence allowing a fast determination of the contamination in the sample. However, when going into the further characterization, due to the loss in absolute numbers of total reads and reads corresponding to *S. aureus*, some enterotoxin encoding genes were missing from the stop_receiving reads. We therefore showed that strain-level characterization was only possible when using all the sequenced reads of the ONT AS approach. Moreover, this level of information could also not be obtained after shotgun metagenomics, demonstrating the added value of using ONT AS. In terms of databases for ONT AS, although each of the used databases was successful for the detection of the pathogen, we showed that the depletion for potato and enrichment for a database of several *S. aureus* genomes obtained better results for a full characterization of the contaminant than an enrichment for a variety of foodborne pathogens. This is probably explained by the dissimilarity of the spiked strain to the only one *S. aureus* genome present in the foodpath database, as was described previously (Martin et al., 2022). The depletion of the matrix, although successfully used for human samples (Cheng et al., 2022; Marquet et al., 2022) and also demonstrated in our study, is a promising pathogen agnostic approach but might however not be practical when set in routine practice, as a database would have to be made for each different sample type, and the composition of the food matrix is not always fully known. Moreover, each genome has to be downloaded before starting the analysis and then included in the database. ONT describes a size limit to the file that can be uploaded for adaptive sampling, and eukaryotic genomes are much larger than microbial genomes, so this might complicate the analysis. Therefore it was determined that a database of the pathogen was the best option for our case study with a known/suspected foodborne contaminant. Having a closely related genome in the database increases the accuracy of the adaptive sampling (Martin et al., 2022), allowing a better downstream characterization of the pathogen. In our study case, the *S. aureus* database contained sequences from two isolates from human origin linked to the outbreak and the strain spiked in the sample, which are all similar. Adding the outbreak strain to the database would be feasible in a real situation when a good communication network (or a shared database) between laboratories allows a rapid sharing of the human pathogen sequences once obtained. Indeed, in case of foodborne outbreaks, if available, the human samples are usually analyzed before the food samples. Therefore, a whole genome sequencing of isolates from human cases, which are more easily obtained, could be conducted and the sequences added in the database used for adaptive sampling. If no human isolate is available, which would make phylogenetic analysis already less relevant, the characterization would benefit from using AS with depletion of the food matrix or enrichment with an extensive database of the pathogen species under investigation, covering the widest possible range of diversity.

Once the settings for the most optimal ONT AS had been determined, the conditions to prepare suitable DNA for ONT AS were tested using mashed potatoes spiked with the living *S. aureus* strain at a level of 10^5^ CFU in 25 grams, representative of a real contamination. ONT AS requires a minimal read length in order to discriminate if the DNA strand is similar or not to the sequences in the database (Buytaers et al., 2022). Moreover, it could be anticipated that host DNA removal prior to AS ONT might even increase the enrichment of target pathogen DNA during ONT AS, avoiding the need of using an ONT AS food matrix depletion approach. Therefore, three DNA extraction kits were tested, including one kit specifically for extraction of high molecular weight DNA (Gand et al., 2023) and one kit depleting the eukaryotic DNA. Using the database of *S. aureus* or foodborne pathogens, we showed a clear influence of the choice of the DNA extraction kit on the results depending on the situation and aim, and the level of characterization desired. The DNA extracted with eukaryotic DNA depletion showed 100 times more reads classified as *S. aureus* after taxonomic classification regardless of the ONT AS database used, therefore leading to an identification of the pathogen within the sample. The capacity of microbial DNA enrichment protocols including the HostZERO kit and the ONT AS were previously described in another study on human intestinal biopsies and the same impact could be observed (Marchukov et al., 2023). However, the same DNA extraction kit did not provide sufficiently good results at strain level to resolve an outbreak. We also observed that less genes were detected after HostZERO extraction. As stated previously (Buytaers et al., 2020), this kit can lead to a biased enhanced detection of genes that are present on plasmids such as in this case *sea, sem, ser*, and *selw* detected in the reconstructed strain (Malachowa and DeLeo, 2010; Vrieling et al., 2020; Nouws et al., 2021). Nonetheless, the two other commercial DNA extraction kits were able to obtain a full characterization of the strain and correct placement in the phylogenetic tree when using the *S. aureus* database. Although one kit was tested for its ability to obtain high molecular weight DNA, no clear advantage during adaptive sampling sequencing and after DNA amplification was noticed, except the strain-level characterization using the more open approach of the foodborne pathogens database, but this result would have to be repeated to be confirmed. Therefore, when the characterization of the pathogen is needed, we propose to use a traditional DNA extraction along with ONT AS using a database of the suspected pathogen when this one is known (based on symptoms, presence of toxins or from analysis of the human cases), and a very fast response is required or if no culture-enrichment is achievable. Especially in the case of toxin-producing pathogens, such as *S. aureus*, our method might be of interest. Toxins are highly resistant to treatments, and therefore the toxin might be present in the suspected food while the producing organism might be inactivated and hence impossible to be isolated. Currently, no method is able to detect all toxins directly in the food sample (Andjelkovic et al., 2016; Lefebvre et al., 2021) and the investigations of a large panel of toxins can only be based on their production in a culture medium after isolation of the bacterial strain (Lefebvre et al., 2022). Metagenomics sequencing of the complete food sample might be the only possibility to screen for the potential of toxin production, as well as obtaining relatedness information to human cases, and hence would contribute to the outbreak investigation when no isolate is available. When the contaminating species is unknown and a fast characterization is necessary, although a culture enrichment might be preferred, the ONT AS with a more open database of a mix of several pathogens can be used to detect some genes or virulence factors on the strain but performing a phylogeny will not be precise enough to place the strain within the outbreak cluster. Alternatively, the matrix depletion during DNA extraction associated with ONT AS using the foodborne pathogens database or the matrix database might be used as a first analysis for orientation, followed by an ONT AS using a specific database of the detected pathogen.

To the best of our knowledge, this level of characterization for a foodborne pathogen without culture-based enrichment presented in this work had never been reported previously. The combination of whole genome amplification, adaptive sampling and metagenomics showed interesting potential in the work presented and was proposed as a first proof of concept to open the way to strain-level metagenomics characterization without enrichment using adaptive sampling. However, its successful implementation in the context of the investigation of foodborne contaminations will still require consideration and optimization of various factors in the future. First of all, the limit of detection of the method will have to be carefully determined, and this should be tested on various matrices and bacterial species. Omitting the culture-based enrichment might lead to false negative results for low levels of contaminations. This could be reduced by combining the taxonomic classification to the detection of species/strain-specific virulence factors (Grützke et al., 2019). Moreover, we acknowledge that although this study included a duplicate of one of the case studies, more replications are necessary. Also, we could observe in this work a strong variation in sequencing output, which was observed even in sequencing repeats and therefore not connected to the DNA input. This was already reported in previous studies (Tyler et al., 2018) and could be due to the variability in number and activity of the nanopores of the different flow cells. In our study, a relation could be noted in some cases between the total number of reads sequenced and the results obtained at strain-level. Indeed, the second repeat of the shotgun sequencing allowed to detect more enterotoxin genes than the first sequencing that generated less reads. The difference in the genes that could be detected is explained by the very low depth of the strain of interest in the shotgun sequencing, which was improved when using adaptive sequencing. Therefore, the stability of the results obtained by ONT sequencing should be further investigated for very low contamination levels (low depth of the strain of interest) using more replicates. We showed that this method works for *S. aureus*, a pathogen with infective dose of log 5 or higher, and similar work could be interesting to conduct on pathogens with similar infective doses such as *B. cereus*, *C. perfringens* or *L. monocytogenes*. However, it is important to note that other frequent foodborne pathogens have a lower infective dose and might not be detected without enrichment, such as *Salmonella*, *Campylobacter* or shiga toxin-producing *E. coli*. Besides testing other pathogens, and hence also other food matrices, it would also be interesting to investigate the feasibility of using adaptive sampling for the scenario where more than one strain of the same pathogenic species is present in the food sample. Co-infection with more than one strain of the same species or genus in the food has been reported in some foodborne outbreaks (Kinnula et al., 2018; Somerville et al., 2018). However, since current methods rely on the analysis of isolates and most often only one food isolate is selected for further characterization, co-contamination of the food sample is very rarely detected using isolation-based methods. We have previously shown that a shotgun metagenomics analysis allows to detect and characterize more than one strain of the same species present in a food sample after culture enrichment of the samples (Buytaers et al., 2020; Saltykova et al., 2020). However, if no culture enrichment is conducted, based on our results, adaptive sampling could be evaluated with a database targeting the pathogen as rich as possible in various strains, covering the full range of possible virulence genes of the species. Alternatively, a depletion of the matrix could also be tested. Furthermore, metagenomic data analysis can be complex and computationally demanding (Escobar-Zepeda et al., 2015). Overall, the costs associated with sequencing, computational resources, data storage and trained personnel can pose challenges, particularly for resource-limited settings or smaller food safety laboratories (Ko et al., 2022). Although the tools proposed in this paper attained the characterization that was required, they also have drawbacks. Indeed, Kraken is known for its relatively high rate of false positives, especially when looking for pathogens at very low levels such as 0.1% of the reads. Furthermore, other possible pathogens were also detected at low percentages, in particular when using a eukaryotic DNA depletion kit, probably due to misclassification as previously shown for low abundance species, reported on long reads with kraken (Portik et al., 2022). In order to make sure that a detected potential pathogen is really present in the sample, downstream analysis could be performed, e.g., mapping of the reads to a reference genome of the pathogen. Other tools might also be used in order to determine the bacterial species in the sample in combination with or in replacement of Kraken (Portik et al., 2022; Gand et al., 2023). Moreover, Metamaps allows strain-level classification of the reads but the tool is tedious to modify the database in order for example to add strains linked to the outbreak. In the future, collaborative efforts within the scientific community are still essential to develop new analysis tools, robust and easy to use frameworks, to enrich databases with new genomes, but also to present guidelines for the effective application of metagenomics within outbreak investigations. Indeed, as this is still an evolving field, some harmonization is necessary, such as the sample preparation protocol or the definition of specific cut-off values within the data analysis. Finally, it is important to note that all sequencing within this study was conducted on R9 flow cells, which is now replaced by R10 flow cells. This technology is evolving quickly, and is expected to give more accurate results with every new release.

In conclusion, we presented for the first time a proof of concept for a method to study food-borne contaminations to the strain-level without the need for culture-based enrichment and isolation, using whole genome amplification and adaptive sampling on metagenomics samples. We showed that metagenomics with ONT AS using a database of foodborne pathogens offers a rapid detection of a microbiological contaminant in the sample while using a database only of the pathogen of interest, here *S. aureus*, allows a full characterization of the pathogen, until the strain level, including the detection of important genes and phylogeny. Determining the presence of a pathogen and further characterizing it without culture enrichment or isolation will contribute to more efficient and accurate microbial food investigations, facilitating timely interventions in case of outbreak and protecting public health.

## Data availability statement

The data presented in this study has been deposited in the NCBI SRA repository, accession numbers under BioProject PRJNA1029022.

## Author contributions

FB: Conceptualization, Data curation, Formal Analysis, Investigation, Methodology, Resources, Software, Visualization, Writing−original draft, Writing−review and editing. BV: Data curation, Resources, Validation, Writing−review and editing. TV: Resources, Writing−review and editing. NR: Funding acquisition, Project administration, Resources, Writing−review and editing. KV: Resources, Software, Writing−review and editing. KM: Resources, Writing−review and editing. SD: Conceptualization, Data curation, Formal Analysis, Funding acquisition, Investigation, Methodology, Project administration, Resources, Supervision, Validation, Writing−original draft, Writing−review and editing.
